# Supplementary material for: Changes in Japanese physicians’ relationships with the pharmaceutical industry between 2008 and 2021: A national survey
Source: PLoS One. 2023 Jun 1;18(6):e0286339. doi: 10.1371/journal.pone.0286339 (PMC10234538; doi:10.1371/journal.pone.0286339)
Supplement: S1 File — (DOCX) [file pone.0286339.s001.docx]

**S1. The survey instrument in English**

1. If you agree to participate in the survey, please check (✔) the box and begin answering.

□　I agree.

1. On average, how often do you experience the following events? Please select one.
   1. Meet with a pharmaceutical representative (PR) face-to-face.

| 1. Never 2. Once a month or less 3. 2-3 times a month 4. Once a week  5. 2-3 times a week 6. More than 4 times a week |
| --- |

- 1. Talk with a PR online.

| 1. Never 2. Once a month or less 3. 2-3 times a month 4. Once a week  5. 2-3 times a week 6. More than 4 times a week |
| --- |

- 1. Exchange emails with a PR．

| 1. Never 2. Once a month or less 3. 2-3 times a month 4. Once a week  5. 2-3 times a week 6. More than 4 times a week |
| --- |

- 1. Receive stationery such as pens from a pharmaceutical company

| 1. Never 2. Once a month or less 3. 2-3 times a month 4. Once a week  5. 2-3 times a week 6. More than 4 times a week |
| --- |

- 1. Receive a medical textbook from a pharmaceutical company.

| 1. Never 2. Once a month or less 3. 2-3 times a month 4. Once a week  5. 2-3 times a week 6. More than 4 times a week |
| --- |

- 1. Participate in a pharmaceutical promotional meeting with meals at the workplace．

| 1. Never 2. Once a month or less 3. 2-3 times a month 4. Once a week  5. 2-3 times a week 6. More than 4 times a week |
| --- |

- 1. Receive meals provided by a pharmaceutical company outside of the workplace.

| 1. Never 2. Once a month or less 3. 2-3 times a month 4. Once a week  5. 2-3 times a week 6. More than 4 times a week |
| --- |

- 1. Participate in webinars involving pharmaceutical companies.

| 1. Never 2. Once a month or less 3. 2-3 times a month 4. Once a week  5. 2-3 times a week 6. More than 4 times a week |
| --- |

1. Please choose the most appropriate answer for each of the following items regarding the informational value of pharmaceutical representatives.
2. PRs play an important role in CME for practicing physicians.

| 1. Agree 2. Somewhat agree 3. Neutral 4. Somewhat disagree 5. Disagree |
| --- |

1. PRs provide accurate information about new medications.

| 1. Agree 2. Somewhat agree 3. Neutral 4. Somewhat disagree 5. Disagree |
| --- |

1. PRs provide accurate information about old (established) medications.

| 1. Agree 2. Somewhat agree 3. Neutral 4. Somewhat disagree 5. Disagree |
| --- |

1. Please choose the most appropriate answer for each of the following items regarding the appropriateness of the physician-industry relationship.
2. Do you think it is ethically appropriate to receive stationery such as pens from pharmaceutical companies?

| 1. Appropriate, 2. Somewhat appropriate, 3. Neutral,  4. Somewhat inappropriate, 5. Inappropriate. |
| --- |

1. Do you think it is ethically appropriate to receive medical textbooks from pharmaceutical companies?

| 1. Appropriate, 2. Somewhat appropriate, 3. Neutral,  4. Somewhat inappropriate, 5. Inappropriate. |
| --- |

1. Do you think it is ethically appropriate to participate in a promotional meeting with meals in the workplace?

| 1. Appropriate, 2. Somewhat appropriate, 3. Neutral,  4. Somewhat inappropriate, 5. Inappropriate. |
| --- |

1. Do you think it is ethically appropriate to receive meals from pharmaceutical companies outside the workplace?

| 1. Appropriate, 2. Somewhat appropriate, 3. Neutral,  4. Somewhat inappropriate, 5. Inappropriate. |
| --- |

1. Please select the most appropriate answer for each of the following items regarding the influence on prescribing behaviors:
2. Do you think that talking about drugs with MR has a negative impact on your prescribing behavior?

| 1. Considerable impact, 2. Some impact, 3. Neutral, 4. Little impact, 5. No impact. |
| --- |

1. Do you think that receiving stationery such as pens has a negative impact on your prescribing behavior?

| 1. Considerable impact, 2. Some impact, 3. Neutral, 4. Little impact, 5. No impact. |
| --- |

1. Do you think that receiving meals from pharmaceutical companies has a netative impact on your prescribing behavior?

| 1. Considerable impact, 2. Some impact, 3. Neutral, 4. Little impact, 5. No impact. |
| --- |

1. Please answer the following items ①-③ regarding pharmaceutical companies and the workplace environment:
2. Do you use items with the name of a pharmaceutical company on them (e.g. pens, notepads) during consultations?

| 1. Yes 　 2. No |
| --- |

1. Are items with the name of a pharmaceutical company (e.g. calendars, organ models) placed in the examination room, waiting room, or waiting corridor of the medical institution where you work?

| 1. They are placed. 　 　2. They are not placed. 　　3. I don’t know |
| --- |

1. Do PRs visit the medical institution where you work?

| 1. Yes, they do visit. 2. No, they don't visit. 3. I don't know. |
| --- |

1. Now I would like to ask about yourself.
2. Gender 　　　　　　　　　1. Male 　 　2. Female 　　3. Other
3. Age　　　　　　　　　　　　　　　　　　　　　years old
4. Year of graduation from medical school: AD　　　　　　 year
5. Practice settings

| 1. Office 2. Public hospital 3. Private hospital  4. Other |
| --- |

1. Specialty

| 1. Internal medicine 2. General surgery 3. Orthopedic surgery  4. Pediatrics 5. Obstetrics/Gynecology 6. Psychiatry 7. Ophthalmology 8. Other |
| --- |

1. Are there rules banning gifts from PRs and/or meetings with PRs in your current workplace?

| 1. There are rules banning both gifts from and meetings with PRs 2. There are rules banning gifts from PRs, but not meetings with PRs 3. There are rules banning meetings with PRs, but not gifts from PRs 4. There are no rules banning both gifts from and meetings with PRs 5. I don’t know. |
| --- |

1. Please answer this question only if you selected 1. or 3. in the question 7.f above.　　　　　　　　　　　　　　　　　　　　　Is the rules banning meetings with PRs for the purpose of preventing the spread of COVID-19 infection?

| 1.Yes 　 2. No 　 3. I don’t know |
| --- |

That conclude the survey. Thank you for your valuable time.
