# Supplementary material for: Changes in Japanese physicians’ relationships with the pharmaceutical industry between 2008 and 2021: A national survey
Source: PLoS One. 2023 Jun 1;18(6):e0286339. doi: 10.1371/journal.pone.0286339 (PMC10234538; doi:10.1371/journal.pone.0286339)
Supplement: S1 Table — Abbreviation: PR, pharmaceutical representative. †Multivariable ordinal logistic regression adjusted for the survey year (2008 or 2021), sex, and clinical setting (clinic or hospital). (DOCX) [file pone.0286339.s002.docx]

**S2 Table. Physician-industry relationships compared with the 2008 survey**

|  | Year | None | <=1/month | 2-3/month | 1/week | 2-3/week | almost every day | *P*-value^†^ |
| --- | --- | --- | --- | --- | --- | --- | --- | --- |
| Meeting with PRs in person (%) | 2008 | 1.7 | 7.7 | 20.2 | 23.5 | 38.0 | 8.9 | < .001 |
|  | 2021 | 21.4 | 31.5 | 24.4 | 10.8 | 10.4 | 1.5 |  |
| Accepting stationery (%) | 2008 | 4.3 | 48.9 | 32.5 | 10.4 | 3.5 | 0.4 | < .001 |
|  | 2021 | 74.3 | 22.8 | 2.4 | 0.3 | 0.1 | 0.1 |  |
| Accepting meals (%) | 2008 | 50.6 | 46.5 | 2.6 | 0.3 | 0 | 0.1 | < .001 |
|  | 2021 | 95.5 | 4.4 | 0.1 | 0 | 0 | 0 |  |

Abbreviation: PR, pharmaceutical representative.

†Multivariable ordinal logistic regression adjusted for the survey year (2008 or 2021), gender, and clinical settings (clinic or hospital).
