# Supplementary material for: Changes in Japanese physicians’ relationships with the pharmaceutical industry between 2008 and 2021: A national survey
Source: PLoS One. 2023 Jun 1;18(6):e0286339. doi: 10.1371/journal.pone.0286339 (PMC10234538; doi:10.1371/journal.pone.0286339)
Supplement: S2 Table — Abbreviation: PR, pharmaceutical representative. †Multivariable ordinal logistic regression adjusted for the survey year (2008 or 2021), sex, and clinical setting (clinic or hospital). (DOCX) [file pone.0286339.s003.docx]

**S3 Table. Physicians' attitudes compared with the 2008 survey**

|  | Year | Agree | Somewhat agree | Neutral | Somewhat disagree | Disagree | *P*-value^†^ |
| --- | --- | --- | --- | --- | --- | --- | --- |
| PRs play an important role in CME for practicing physicians. (%) | 2008 | 29.4 | 44.1 | 14.8 | 9.3 | 2.5 | < .001 |
|  | 2021 | 24.3 | 41.8 | 18.6 | 10.9 | 4.4 |  |
| PRs provide accurate information on new medications. (%) | 2008 | 17.7 | 55.4 | 18.7 | 6.4 | 1.8 | 0.06 |
|  | 2021 | 21.4 | 52.8 | 17.8 | 6.2 | 1.7 |  |
| PRs provide accurate information on established medications. (%) | 2008 | 7.7 | 38.1 | 33.2 | 16.4 | 4.5 | 0.22 |
|  | 2021 | 10.0 | 33.8 | 30.6 | 19.0 | 6.5 |  |
| Meeting with PRs has an impact on my prescribing behaviors. (%) | 2008 | 0.8 | 4.9 | 25.4 | 44.4 | 24.5 | 0.0015 |
|  | 2021 | 1.6 | 12.5 | 17.6 | 48.9 | 19.4 |  |

Abbreviation: PR, pharmaceutical representative.

† Multivariable ordinal logistic regression adjusted for the survey year (2008 or 2021), gender, and clinical settings (clinic or hospital).
