# Supplementary material for: Changes in Japanese physicians’ relationships with the pharmaceutical industry between 2008 and 2021: A national survey
Source: PLoS One. 2023 Jun 1;18(6):e0286339. doi: 10.1371/journal.pone.0286339 (PMC10234538; doi:10.1371/journal.pone.0286339)
Supplement: Attachment — Submitted filename: Response to Reviewers.docx [file pone.0286339.s004.docx]

Reviewer #1: I think its important think about the pharmaceutical sales. Pharmaceutical representatives doing a good job to promote their companies product. How they can convince the doctor to increase the sale of their product is important. I am excited that the Japanese specialty doctors are not influenced by the promotions given by the PRs. However, the study does not seem novel.

Response: Thank you for your review. As you noted, there have been numerous studies conducted on this topic in the past. However, this particular study is significant as it sheds light on the fact that the perception of Japanese physicians has not changed over time, despite long-standing concerns. We believe that this finding is a valuable contribution to the existing body of knowledge on physician-pharmaceutical industry relationships.

Reviewer #2: The article discusses the involvement of physician in pharmaceutical promotional activities and attempts to track the changes from a previous 2008 study. They used a survey tool that partly resembles their previous tool which makes it hard to track this change. Also the survey tool was not part of the publication, not knowing the language of the question makes it hard to understand the responses provided.

Response: Thank you for your comment. We have added a survey form in which the questions are in English as a supplementary file.

Reviewer #2: There was no logical explanation on why different specialties was a determining factor for significance.

Response: Since previous studies have indicated that physicians who prescribe more also receive more gifts, we compared the differences by specialty and expected that internists, who are known to prescribe more, would be more involved in pharmaceutical promotional activities than other specialists. We have added an explanation of this point on page 7, line 19 to page 8, line 3 of the manuscript.

Reviewer #2: In addition, unlike the research done on similar topics in the US, there is no legislation or decision from professional organizations or medical schools that deters physician from interacting/accepting gifts and promotional material from pharmaceutical representative, so the variable studied between 2008 and now is, and based on the study findings, not a determining factor to change attitudes/behaviors.

Response: We agree that the variable studied between 2008 and now is not a determining factor to change attitudes or behaviors, as per the study findings. We have added an explanation of this point on page 25 lines 3 to 7 of the manuscript.
